# Efficacy of limaprost combined with unilateral biportal endoscopic surgery in the treatment of lumbar spinal stenosis: based on time effects and stratified analysis

**DOI:** 10.3389/fsurg.2026.1869975

**Published:** 2026-06-25

**Authors:** Yongda Yue, Shumei Chen, Jinguo Chen, Xiande Lin, Junyi Chen, Weiting Lin, YuanBiao Luo

**Affiliations:** 1 Orthopedics, The First Hospital of Putian, Putian, China; 2 Department of Pathology, The First Hospital of Putian, Putian, Fujian, China

**Keywords:** efficacy evaluation, generalized estimating equations (GEE), limaprost, lumbar spinal stenosis (LSS), stratified analysis, unilateral biportal endoscopic (UBE) surgery

## Abstract

**Background:**

Lumbar spinal stenosis (LSS) is a common degenerative spinal disease, frequently observed in the elderly population, characterized by symptoms such as low back pain and radiating pain in the lower extremities, which significantly impairs quality of life.

**Methods:**

Patients were divided into two groups according to treatment modality: the intervention group (limaprost combined with UBE surgery) and the control group (UBE surgery alone). At baseline and at 1 week, 1 month, 3 months, and 6 months postoperatively, both groups were evaluated using the Visual Analog Scale (VAS), Oswestry Disability Index (ODI), Japanese Orthopaedic Association (JOA) score, modified MacNab criteria, Manual Muscle Testing (MMT), cross-sectional area (CSA), and anteroposterior diameter of the spinal canal. A generalized estimating equation (GEE) model was used to analyze the interaction effect between treatment method and follow-up time on the above outcomes. A multiple linear regression model was applied to assess the effects of the intervention in different patient subgroups.

**Results:**

After treatment, the intervention group showed significantly better results in the primary outcome (VAS) and secondary outcomes (ODI, JOA, MMT, and modified MacNab scores) compared with the control group, whereas no significant improvement was observed in CSA or the anteroposterior diameter of the spinal canal. The intervention demonstrated a stronger effect on pain relief at 1 month postoperatively and a more pronounced effect on neurological function recovery at 3 months postoperatively; however, the improvement in functional disability did not change over time. Subgroup analysis indicated that patients aged ≥65 years, with BMI < 25, and with central-type stenosis were more suitable for treatment with limaprost combined with UBE surgery.

**Conclusion:**

These findings suggest that in the postoperative management of LSS patients, the combined application of limaprost and UBE surgery was associated with better clinical outcomes, particularly in pain reduction and neurological recovery, thereby potentially providing a new reference for the selection and optimization of related treatment strategies.

## Introduction

Lumbar spinal stenosis (LSS) is a degenerative disease characterized by narrowing of the lumbar spinal canal, which may involve the central canal, lateral recess, and intervertebral foramina, subsequently leading to compression of neural structures (1–3). Its development is commonly associated with multiple degenerative changes, including intervertebral disc degeneration, ligamentum flavum hypertrophy, facet joint hypertrophy, and osteophyte formation at the posterior vertebral margin (4). Epidemiological studies have shown that 19%–47% of individuals over 60 years of age present with anatomical spinal stenosis (5). Clinically, it is mainly manifested by low back and lower limb pain, limited walking ability (typically intermittent claudication), sensory abnormalities, and muscle weakness in the lower extremities (5). In severe cases, varying degrees of neurological impairment may occur. In addition to its physical impact, LSS also affects patients' mental health; for example, E Özdemir et al. reported that anxiety and depression scores in LSS patients were significantly higher than those in the control group (6). With the continuous progression of population aging, the number of patients with LSS is increasing (7). Although conservative treatment can provide symptom relief in the early stage of the disease, surgical decompression remains a key intervention for relieving neural compression and improving functional outcomes in patients with moderate to severe disease (8).

In recent years, with the rapid development of minimally invasive spinal surgical techniques, unilateral biportal endoscopy (UBE), as a novel spinal endoscopic technique, has been widely applied in the surgical treatment of degenerative lumbar diseases (9–11). UBE offers advantages such as a wide field of view, large working space, minimal tissue injury, and rapid postoperative recovery, making it particularly suitable for patients with lumbar spinal stenosis requiring bilateral or multilevel decompression. However, UBE primarily focuses on mechanical decompression, and some patients still experience residual neurological dysfunction and impaired microcirculatory perfusion after surgery. Based on this, further optimization of treatment outcomes and improvement of long-term prognosis not only facilitate neurological recovery but may also reduce the risks of restenosis and reoperation, thus holding important clinical significance.

At present, many studies have reported the efficacy of UBE in the treatment of LSS (12–14), however, research on the combination of limaprost and UBE remains limited. This study aims to fill this gap by further analyzing the effects of limaprost combined with UBE surgery at different time points and in different patient populations, thereby providing more scientific treatment strategies for patients with LSS.

## Materials and methods

### Study population

This retrospective study included patients with LSS who underwent UBE surgery at our hospital between January 2023 and January 2024. The inclusion criteria were: 1) age ≥18 years; 2) meeting the diagnostic criteria for LSS; 3) eligible for UBE surgery; and 4) complete clinical data available. The exclusion criteria were: 1) concomitant lumbar disorders; 2) pregnancy or lactation; 3) missing critical data; 4) contraindications to limaprost; 5) severe systemic diseases such as malignancy or advanced-stage cancer; and 6) severe psychiatric disorders (including schizophrenia spectrum disorders, mood disorders, intellectual disability, or developmental disorders) or poor treatment adherence. After applying the above inclusion and exclusion criteria, a total of 100 patients were enrolled and were divided into the control group and intervention group based on the actual treatment received.

### Intervention

Control Group: All patients underwent general anesthesia and preoperative catheterization. Patients were placed in the prone position, and the C-arm was used to adjust the operating table so that the intervertebral space at the surgical segment was as perpendicular to the ground as possible. Preoperative C-arm fluoroscopy was performed for surface localization to confirm the surgical segment and intervertebral space. The disc level line, as well as the line connecting the inner edges of the adjacent vertebral pedicles on the symptomatic surgical side, was marked to identify the endoscopic observation channel and the surgical working channel. The endoscopic observation channel was located approximately 1 cm above or below the intervertebral disc level line, and the surgical working channel was located approximately 2 cm above or below the intervertebral disc level line. After disinfection and sterile draping, fluid circulation for water-based endoscopy was established. A 1 cm incision was made at the marked observation channel site, and a dilator and working cannula were inserted. A 1.5 cm incision was made at the working channel site, followed by insertion of the working cannula. C-arm fluoroscopy in both anteroposterior and lateral views was used to confirm correct positioning of the cannulas. The endoscope was connected, with the left hand holding the endoscope and the right hand performing the operation. The posterior interlaminar space of the segment was exposed under endoscopic visualization. Specialized drills were used to remove the inferior articular process to expose the ligamentum flavum and the superior articular process. A bony window was created by removing bone to the head, tail, and lateral margins of the ligamentum flavum. The base of the spinous process and the contralateral facet process were also removed to expose the contralateral superior articular process. Under endoscopic visualization, a bone chisel was used to remove part of the contralateral superior articular process, exposing the contralateral nerve root lateral recess. All ligamentum flavum was removed to expose the dura and bilateral nerve roots, which were then decompressed. Disc exploration was performed to check for protrusion. After confirming adequate decompression of the head, caudal, and bilateral edges of the spinal canal, hemostasis was achieved under endoscopy. A drainage tube was placed in the working incision, and the wound was closed.

Intervention Group: The intervention group received oral limaprost in addition to the control group treatment, at a dose of 1 tablet (5 μg) three times daily. Each box contains 10 tablets, which is sufficient for approximately 3 days of use. A total of 17 boxes were administered, covering the entire perioperative period, corresponding to an overall treatment duration of approximately 51 days (17 boxes × 10 tablets ÷ 3 doses per day). This medication regimen was applied throughout the perioperative management period. Medication adherence was assessed through outpatient follow-up records and medical chart review. Patients with adherence below 80% were excluded according to exclusion criterion No. 6 (poor treatment adherence). Therefore, the included patients in this study demonstrated generally good overall medication adherence.

### Data collection

Patients' demographic and clinical information, including age, gender, BMI, and lifestyle habits, was obtained through questionnaires and clinical assessments by the attending physicians. Imaging data, including the levels involved and imaging-based classification, were acquired via magnetic resonance imaging (MRI). Assessments were performed at pre-treatment (T0), postoperative 1 week (T1), 1 month (T2), 3 months (T3), and 6 months (T4), including Pain Visual Analogue Scale (VAS), Oswestry Disability Index (ODI), Japanese Orthopaedic Association score (JOA) (15), Manual Muscle Testing (MMT), Modified MacNab Criteria, and imaging indicators such as dural sac cross-sectional area (CSA) and spinal canal anteroposterior diameter. All assessments were conducted by trained researchers to ensure data reliability and consistency.

The primary outcome was the VAS score. Secondary outcomes included the ODI, JOA score, modified MacNab criteria, Manual Muscle Testing (MMT), and radiological parameters (cross-sectional area and anteroposterior diameter of the spinal canal).

During the actual follow-up period, a small amount of loss to follow-up and missing data occurred at each time point, with the proportion being less than 10%. For these missing data, multiple imputation was applied in the group comparisons and multivariable linear regression analyses. Since the longitudinal model (GEE) is robust to incomplete follow-up data and can utilize all available observations for estimation, no additional imputation was performed for this analysis.

## Generalized estimating equations (GEE)

The group (intervention vs. control) and time points (T0, T1, T2, T3, T4) were treated as fixed-effect variables, and VAS, ODI, and JOA scores were used as dependent variables to construct models including the interaction term (Group   ×   Time). The models employed a repeated-measures design with an exchangeable correlation structure to account for correlations between multiple measurements within the same subject. Three models were constructed: Model 1 without adjustment for any covariates, Model 2 adjusted for age, sex, and BMI, and Model 3 further adjusted for age, sex, BMI, affected spinal level, and imaging-based classification, in order to evaluate whether the interaction between treatment and time was influenced by covariates.

### Stratified multivariate linear regression analysis

The changes in VAS, ODI, and JOA scores were used as dependent variables, calculated as the difference between the 6-month postoperative scores (T4) and the pre-treatment scores (T0). The independent variables included the treatment method, age, sex, BMI, affected spinal level, and imaging-based classification. Patients were stratified by age (<65 years vs. ≥ 65 years), sex (male vs. female), BMI (<25 vs. ≥ 25), and imaging-based classification (central canal stenosis, lateral recess stenosis, combined stenosis). Within each stratum, separate multivariate regression models were constructed to evaluate the effect of the treatment method on the change in scores (regression coefficient B and 95% confidence interval), and the corresponding statistical significance (*p*-value) was calculated. Visualization of the results was subsequently performed.

### Statistical analysis

All analyses and visualizations in this study were performed using R software version 4.4.1. Continuous variables were expressed as median (minimum–maximum) and compared using independent-samples *t*-test or Mann–Whitney *U* test, as appropriate. Categorical variables were presented as counts and percentages (*n*, %) and compared between groups using the *χ*² test or Fisher's exact test. All tests were two-sided, and a *p*-value < 0.05 was considered statistically significant. A *post hoc* statistical power analysis was performed to evaluate the statistical efficiency of the sample size. Under a significance level of *α* = 0.05 and an effect size of d = 0.5 (moderate effect), with a statistical power of 0.70, the required sample size was approximately 50 participants per group. To control for baseline differences between groups and reduce the influence of potential confounding factors, propensity score matching (PSM) was further performed as a sensitivity analysis in this study. All baseline characteristics were included as covariates. A 1:1 nearest-neighbor matching method was applied with a caliper value of 0.1. After matching, covariate balance was assessed using standardized mean differences (SMDs), with an SMD < 0.1 generally considered indicative of adequate covariate balance.

## Results

### Baseline characteristics of the control and intervention groups

The results showed that the control and intervention groups were comparable in terms of age, gender, BMI, and lifestyle factors, with no statistically significant differences between the two groups (*P* > 0.05). Similarly, disease-related characteristics and imaging features were also comparable, with no significant differences observed (*P* > 0.05). Overall, the two groups demonstrated good baseline comparability (Table 1). After PSM, the SMDs of most covariates were reduced to below 0.1, and the points converged toward zero, indicating good baseline balance between the two groups (Supplementary Table 1; Supplementary Figure 1).

**Table 1:** Distribution of baseline characteristics between the control and intervention groups.

Characteristic	Overall	Control group	Intervention group	*P*-value
	*n* = 100	*n* = 50	*n* = 50	
Age	65 (54–81)	65 (54–80)	66 (56–81)	0.669
Gender				0.072
Male	48 (48%)	19 (38%)	29 (58%)	
Female	52 (52%)	31 (62%)	21 (42%)	
BMI	25.2 (21.6–28.8)	25.0 (21.7–28.6)	25.2 (21.6–28.8)	0.581
Smoking				0.386
Non-smoker	73 (73%)	36 (72%)	37 (74%)	
Occasional smoker	18 (18%)	11 (22%)	7 (14%)	
Regular smoker	9 (9%)	3 (6%)	6 (12%)	
Drinking				0.080
Non-drinker	66 (66%)	36 (72%)	30 (60%)	
Occasional drinker	23 (23%)	12 (24%)	11 (22%)	
Regular drinker	11 (11%)	2 (4%)	9 (18%)	
Retirement	82 (82%)	40 (80%)	42 (84%)	0.795
Exercise habit				0.751
No	56 (56%)	29 (58%)	27 (54%)	
Occasional	36 (36%)	18 (36%)	18 (36%)	
Regular	8 (8%)	3 (6%)	5 (10%)	
Duration of disease (months)	23 (6–35)	23 (6–35)	23 (7–33)	0.482
Multi-level involvement	56 (56%)	23 (46%)	33 (66%)	0.070
Affected spinal level				
L3-L4	23 (23%)	13 (26%)	10 (20%)	0.635
L4-L5	71 (71%)	32 (64%)	39 (78%)	0.186
L5-S1	17 (17%)	12 (24%)	5 (10%)	0.110
Imaging-based classification				0.189
Central canal stenosis	54 (54%)	30 (60%)	24 (48%)	
Lateral recess stenosis	20 (20%)	11 (22%)	9 (18%)	
Combined stenosis	26 (26%)	9 (18%)	17 (34%)	

### Comparison of functional and radiological outcome measures between the control and intervention groups at pre-treatment, 1 week, 1 month, 3 months, and 6 months postoperatively

At baseline (T0), there were no significant differences between the control and intervention groups in VAS, ODI, JOA, MMT, CSA, or spinal canal anteroposterior diameter. At various postoperative time points, all functional outcome measures significantly improved compared with baseline in both groups, with the intervention group showing superior improvement. Specifically, the intervention group exhibited lower VAS and ODI scores and higher JOA and MMT scores. However, no significant differences were observed between the groups in CSA or spinal canal anteroposterior diameter. In addition, according to the modified MacNab criteria, the proportion of patients rated as “Excellent” in the intervention group was significantly higher than in the control group at 1 month, 3 months, and 6 months postoperatively. After false discovery rate (FDR) correction, some results that were statistically significant in the unadjusted analysis were no longer significant (mainly in the JOA outcomes). Therefore, these findings are interpreted as exploratory and are presented in a descriptive manner only (Table 2). Regarding postoperative complications, both groups demonstrated good safety profiles, with comparable incidence rates. No limaprost related adverse events were observed in the intervention group during the follow-up period (Table 3).

**Table 2:** Differences in functional and radiological outcome measures between the control and intervention groups at preoperative, 1 week, 1 month, 3 months, and 6 months postoperatively.

Outcome	Overall	Control group	Intervention group	*P*-value	FDR
	*n* = 100	*n* = 50	*n* = 50		
Visual Analogue Scale (VAS)					
T0	7 (6–9)	7 (6–9)	7 (6–9)	0.205	0.308
T1	5 (3–6)	5 (3–6)	4 (3–6)	0.014	0.039
T2	3 (1–5)	4 (1–5)	2 (1–5)	0.039	0.078
T3	2 (1–3)	2 (1–3)	2 (1–3)	0.010	0.037
T4	1 (0–2)	1 (0–2)	1 (0–2)	0.006	0.028
Oswestry disability index (ODI)					
T0	54 (45–61)	55 (45–61)	53 (45–61)	0.412	0.523
T1	42 (35–48)	43 (35–48)	40 (35–45)	0.014	0.039
T2	27 (19–34)	30 (19–34)	25 (19–32)	0.001	0.017
T3	19 (12–25)	21 (12–25)	16 (12–22)	0.003	0.020
T4	10 (6–14)	11 (7–14)	8 (6–14)	0.001	0.017
Japanese orthopaedic association(JOA)					
T0	17 (15–19)	17 (15–19)	17 (15–19)	0.162	0.255
T1	20 (19–22)	20 (19–22)	21 (19–22)	0.043	0.078
T2	22 (21–24)	22 (21–24)	23 (21–24)	0.032	0.075
T3	24 (22–26)	24 (22–26)	25 (22–26)	0.002	0.017
T4	25 (23–27)	24 (23–27)	26 (23–27)	0.034	0.075
Manual muscle testing (MMT) (Proportion of grade 5)					
T0	8 (8%)	2 (4%)	6 (12%)	0.269	0.368
T1	18 (18%)	3 (6%)	15 (30%)	0.004	0.022
T2	47 (47%)	18 (36%)	29 (58%)	0.045	0.078
T3	61 (61%)	24 (48%)	37 (74%)	0.014	0.039
T4	86 (86%)	37 (74%)	49 (98%)	0.002	0.017
Modified MacNab criteria (proportion of excellent)					
T2	43 (43%)	16 (32%)	27 (54%)	0.043	0.078
T3	62 (62%)	24 (48%)	38 (76%)	0.007	0.029
T4	77 (77%)	33 (66%)	44 (88%)	0.017	0.043
Dural sac cross-sectional area (CSA) (mm²)					
T0	57.6 (39.1–74.3)	57.1 (39.3–73.2)	59.8 (39.1–74.3)	0.581	0.710
T1	111.7 (91.5–131.3)	110.9 (91.7–130.6)	112.9 (91.5–131.3)	0.983	0.994
T2	122.3 (99.3–145.6)	121.8 (101.5–145.4)	124.0 (99.3–145.6)	0.983	0.994
T3	127.9 (102.0–145.5)	126.9 (102.0–145.4)	128.8 (102.9–145.5)	0.809	0.890
T4	123.0 (104.4–145.5)	121.1 (104.4–145.5)	125.5 (104.6–142.9)	0.644	0.759
Spinal canal anteroposterior diameter (mm)					
T0	7.5 (6.5–9.0)	7.6 (6.5–9.0)	7.5 (6.6–9.0)	0.267	0.368
T1	12.9 (11.5–14.3)	13.0 (11.5–14.3)	12.8 (11.5–14.3)	0.994	0.994
T2	13.5 (12.1–14.8)	13.5 (12.2–14.8)	13.4 (12.1–14.8)	0.705	0.802
T3	13.6 (12.6–14.8)	13.7 (12.6–14.8)	13.5 (12.6–14.8)	0.279	0.368
T4	13.9 (12.5–15.1)	14.1 (12.5–15.1)	13.7 (12.5–15.1)	0.108	0.178

**Table 3:** Incidence of complications in the control and intervention groups.

Complications	Overall	Control group	Intervention group
	*n* = 100	*n* = 50	*n* = 50
Dural tear	1 (1%)	0 (0%)	1 (2%)
Infection	1 (1%)	1 (2%)	0 (0%)
Hematoma	1 (1%)	1 (2%)	0 (0%)

### Effects of the interaction between treatment method and follow-up time on VAS, ODI, and JOA scores

For VAS pain scores, the interaction term coefficients (B) at all postoperative time points (T1–T4) in the three models were negative and statistically significant (*p* < 0.05), indicating that the reduction in VAS scores over time was significantly greater in the intervention group compared with the control group. For ODI scores, the interaction coefficients were also negative across all time points in the three models but did not reach statistical significance. Regarding JOA scores, the interaction coefficients were positive at all time points, with *p*-values < 0.05, suggesting that the improvement in JOA scores over time was significantly greater in the intervention group than in the control group (Table 4).

**Table 4:** GEE analysis of the interaction between treatment method and follow-up time on VAS, ODI, and JOA.

Outcome	Item	Model 1			Model 2			Model 3		
		B	CI	*p*.value	B	CI	*p*.value	B	CI	*p*.value
VAS	Group*TimeT1	−0.638	[−1.103, −0.173]	0.007	−0.638	[−1.100, −0.176]	0.007	−0.638	[−1.098, −0.178]	0.007
	Group*TimeT2	−0.674	[−1.242, −0.105]	0.020	−0.674	[−1.238, −0.110]	0.019	−0.674	[−1.230, −0.117]	0.018
	Group*TimeT3	−0.482	[−0.884, −0.081]	0.019	−0.482	[−0.880, −0.084]	0.018	−0.482	[−0.876, −0.088]	0.016
	Group*TimeT4	−0.527	[−0.933, −0.121]	0.011	−0.527	[−0.929, −0.124]	0.010	−0.527	[−0.925, −0.129]	0.010
ODI	Group*TimeT1	−0.734	[−2.933, 1.465]	0.513	−0.734	[−2.934, 1.467]	0.513	−0.734	[−2.924, 1.457]	0.511
	Group*TimeT2	−2.253	[−4.684, 0.179]	0.069	−2.253	[−4.682, 0.176]	0.069	−2.253	[−4.688, 0.182]	0.070
	Group*TimeT3	−1.551	[−3.889, 0.786]	0.193	−1.551	[−3.882, 0.780]	0.192	−1.551	[−3.872, 0.769]	0.190
	Group*TimeT4	−0.671	[−2.676, 1.335]	0.512	−0.671	[−2.673, 1.332]	0.512	−0.671	[−2.675, 1.334]	0.512
JOA	Group*TimeT1	0.679	[0.138, 1.220]	0.014	0.679	[0.146, 1.212]	0.013	0.679	[0.152, 1.207]	0.012
	Group*TimeT2	0.676	[0.155, 1.198]	0.011	0.676	[0.162, 1.191]	0.010	0.676	[0.168, 1.185]	0.009
	Group*TimeT3	1.003	[0.414, 1.593]	0.001	1.003	[0.422, 1.585]	0.001	1.003	[0.425, 1.582]	0.001
	Group*TimeT4	0.828	[0.204, 1.453]	0.009	0.828	[0.207, 1.449]	0.009	0.828	[0.208, 1.449]	0.009

### Stratified multivariate linear regression analysis of the effect of treatment method on VAS, ODI, and JOA scores

**Figure 1:**
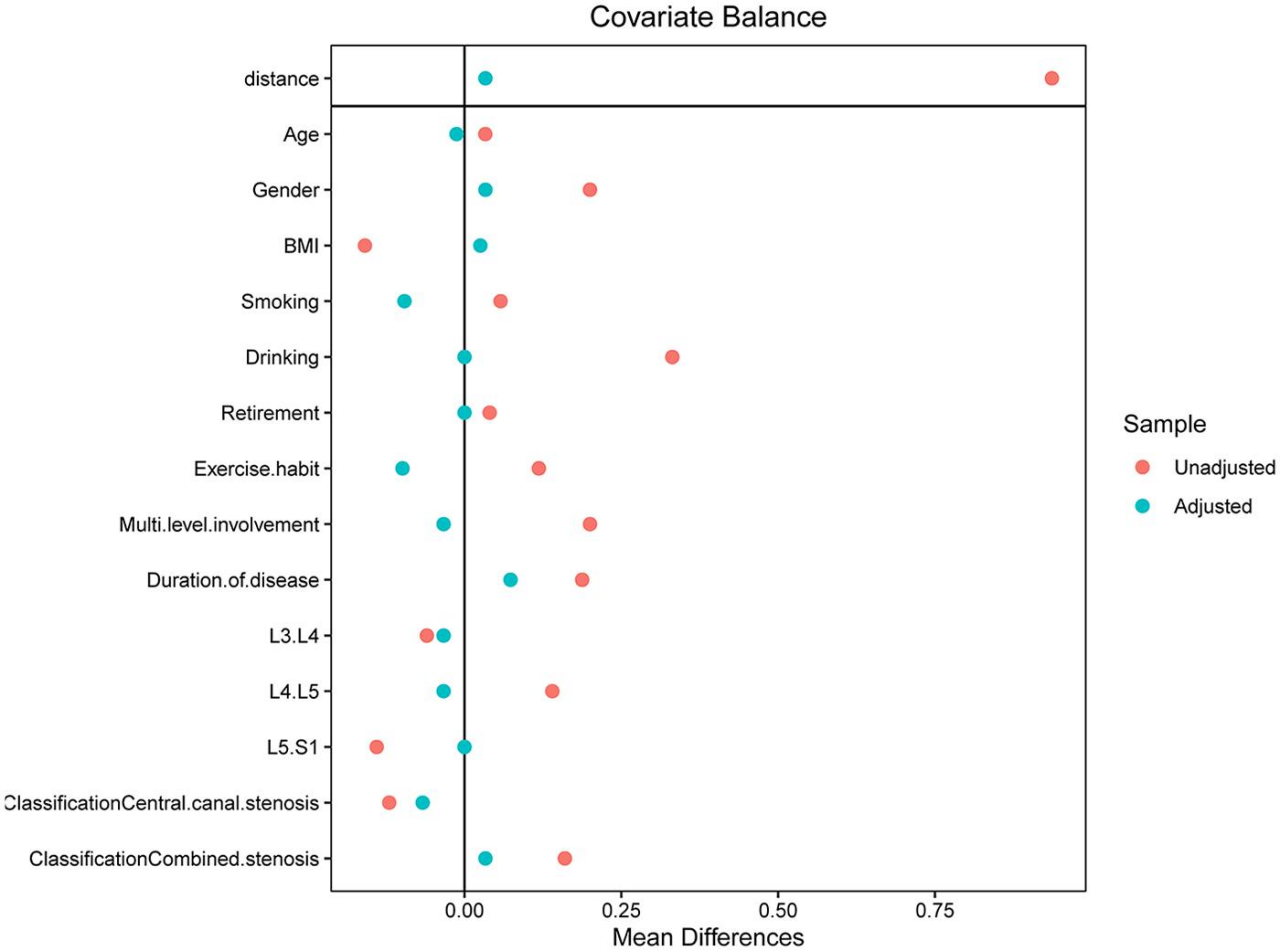
Visualization of stratified multivariate linear regression results.

The results showed that in patients aged ≥65 years, the treatment method was significantly negatively associated with the change in VAS (B = −1.164, *p* < 0.001) and significantly positively associated with the change in JOA (B = 1.377, *p* = 0.002), while the association with ODI change was not statistically significant (*p* = 0.146), indicating that elderly patients benefited more in terms of pain relief and functional recovery. In male patients, the treatment method was significantly negatively associated with VAS change (B = −0.816, *p* = 0.043) and showed a non-significant negative association with ODI change (*p* = 0.051), but no significant association with JOA change (*p* = 0.189). In female patients, the treatment method was significantly positively associated with JOA change (B = 1.636, *p* < 0.001). In patients with BMI <25, the treatment method was significantly associated with VAS change (B = −1.044, *p* = 0.006), ODI change (B = −3.051, *p* = 0.001), and JOA change (B = 0.975, *p* = 0.029); whereas in patients with BMI ≥25, the treatment method was only significantly positively associated with JOA change (B = 1.335, *p* = 0.006). In patients with central canal stenosis, the treatment method showed significant superiority over the control group in improving ODI (*p* = 0.016) and JOA (*p* = 0.001), while no significant differences were observed in lateral recess or combined stenosis subgroups (*p* > 0.05). From an outcome perspective, the treatment method was significantly associated with JOA improvement across all five subgroups, whereas significant associations with ODI and VAS improvements were observed in only two and three subgroups, respectively (Table 5; Figure 1).

**Table 5:** Multivariate linear regression stratified analysis of the effect of treatment method on VAS, ODI, and JOA.

Stratification	VAS change					ODI change					JOA change				
	B	CI	std.error	Statistic	*p*.value	B	CI	std.error	Statistic	*p*.value	B	CI	std.error	Statistic	*p*.value
Age < 65 (*n* = 38)															
Group	−0.042	[−1.056, 0.973]	0.517	−0.080	0.937	−1.756	[−4.031, 0.520]	1.161	−1.512	0.143	1.034	[−0.135, 2.204]	0.596	1.734	0.095
Age >= 65 (*n*=62)															
Group	−1.164	[−1.754, −0.573]	0.301	−3.864	0.000	−1.213	[−2.824, 0.397]	0.822	−1.477	0.146	1.377	[0.552, 2.202]	0.421	3.272	0.002
Male (*n* = 48)															
Group	−0.816	[−1.579, −0.052]	0.390	−2.093	0.043	−1.739	[−3.426, −0.052]	0.861	−2.021	0.051	0.642	[−0.299, 1.584]	0.480	1.338	0.189
Female (*n* = 52)															
Group	−0.281	[−0.957, 0.395]	0.345	−0.814	0.420	−0.931	[−2.736, 0.874]	0.921	−1.011	0.318	1.636	[0.797, 2.474]	0.428	3.823	0.000
BMI < 25 (*n* = 48)															
Group	−1.044	[−1.750, −0.337]	0.360	−2.896	0.006	−3.051	[−4.699, −1.404]	0.840	−3.631	0.001	0.975	[0.137, 1.813]	0.427	2.281	0.029
BMI >= 25 (*n* = 52)															
Group	−0.121	[−0.850, 0.608]	0.372	−0.325	0.747	0.191	[−1.525, 1.907]	0.876	0.218	0.829	1.335	[0.426, 2.245]	0.464	2.877	0.006
Central canal stenosis (*n* = 54)															
Group	−0.121	[−0.850, 0.608]	0.372	−0.325	0.747	−2.061	[−3.676, −0.445]	0.824	−2.500	0.016	1.405	[0.672, 2.137]	0.374	3.757	0.001
Lateral recess stenosis (*n* = 20)															
Group	−0.503	[−1.806, 0.800]	0.665	−0.757	0.468	−0.890	[−3.417, 1.636]	1.289	−0.691	0.507	1.108	[−0.573, 2.790]	0.858	1.292	0.229
Combined stenosis (*n* = 26)															
Group	−0.831	[−1.874, 0.211]	0.532	−1.563	0.139	−0.925	[−3.814, 1.964]	1.474	−0.627	0.540	1.209	[−0.506, 2.924]	0.875	1.382	0.187

## Discussion

The results of this study showed no significant differences between the intervention and control groups in structural imaging indicators, including spinal canal cross-sectional area (CSA) and spinal canal diameter. Regarding this negative finding, one possible explanation is that limaprost, as a pharmacological agent, cannot directly improve spinal canal anatomy, while structural improvement mainly depends on the decompression procedure itself. Since both groups underwent UBE surgery, no significant between-group differences were observed in imaging outcomes. However, improvements in pain and functional outcomes, including VAS, ODI, and JOA scores, were more evident. These findings suggest that limaprost may reduce pain and promote recovery of functional capacity and neurological function through mechanisms such as microvascular dilation and improvement of local blood circulation (16, 17).

Limaprost combined with UBE was associated with better VAS and JOA outcomes in patients aged ≥65 years, whereas this association was not significant in patients aged <65 years. This may be because older patients are more likely to have greater neural ischemic factors. One of the mechanisms of lumbar spinal stenosis (LSS) is that compression of the nerve root microcirculation leads to ischemia (18). Even after surgical treatment, elderly patients may still present a certain degree of chronic ischemia–hypoperfusion status in the early postoperative period. In this context, perioperative treatment with limaprost may improve outcomes, whereas younger patients generally achieve relatively good postoperative recovery, and the additional benefit of limaprost is therefore limited. Intervention group showed better improvement in VAS scores in male patients, while it showed better improvement in JOA scores in female patients. This may be due to sex-related differences in pain perception and expression (19, 20), males are often more sensitive to relief of mechanical compression, and UBE itself reduces pain through mechanical decompression. Together with the microcirculation-improving effect of limaprost, the analgesic effect is more pronounced. Estrogen has neuroprotective effects (21), although estrogen levels in female patients in this study were significantly reduced, it may still have certain effects in processes such as axonal regeneration and myelin repair. Patients with lower BMI showed significant improvements in VAS, ODI, and JOA scores. This may be because such patients have lower axial loading on the lumbar spine and lower intervertebral disc and facet joint pressure. After UBE relieves nerve root compression, the microcirculation-improving effect of limaprost may facilitate pain relief, and daily activity burden is also significantly reduced. In contrast, obese patients often have a chronic low-grade inflammatory state and more pronounced neural sensitization (22), which may limit the improvement in VAS scores. In obese patients, increased axial loading of the lumbar spine persists even after UBE, leaving the nerve roots in an unfavorable mechanical environment, thereby limiting functional recovery as reflected by ODI. Patients with central lumbar spinal stenosis showed better clinical improvement than those with lateral recess stenosis. This may be because central stenosis is mainly characterized by diffuse compression of the dural sac and cauda equina, with a relatively concentrated lesion area, allowing UBE to achieve more sufficient and direct decompression of the central canal and effectively relieve the main compressive factors. In contrast, lateral recess stenosis more often involves a single nerve root canal, with a more focal and spatially restricted anatomy, and a more complex pattern of compression requiring higher precision in decompression. In addition, due to the more distributed arrangement and relatively richer blood supply of the cauda equina, its reversibility to chronic compression may be higher, leading to faster functional recovery after decompression of the central canal. In contrast, nerve roots affected by lateral recess stenosis are more likely to experience localized and sustained compression, with more structural nerve injury, thereby limiting postoperative functional recovery. It should be noted that the above mechanistic interpretations are speculative and based on previous literature. These factors were not directly measured or validated in the present study and should therefore be considered as hypothesis-generating. Future studies incorporating relevant biochemical experiments are needed to further validate these potential pathways.

The GEE results suggested that the intervention was associated with more pronounced changes in VAS scores at T1 (1 week postoperatively) and T2 (1 month postoperatively), whereas more evident changes in JOA scores were observed at T3 (3 months postoperatively). This may be because limaprost's vasodilatory and anti-inflammatory effects can rapidly improve local microcirculation and reduce inflammatory edema in the early postoperative period, thereby alleviating mechanical compression and chemical irritation of the nerve roots, leading to decreased pain levels. Neural functional recovery, on the other hand, mainly depends on processes such as reperfusion of compressed nerve roots, axonal regeneration, and myelin repair, and limaprost's effects on promoting neural regeneration and functional remodeling may require a longer duration to manifest. We noted that ODI showed significant between-group differences in the univariate comparisons at individual time points (Table 2), whereas it was not significant in the GEE analysis. This discrepancy may be explained by the fact that the GEE model evaluates the overall longitudinal interaction effect over time, while accounting for within-subject correlation and repeated-measures structure. Therefore, the GEE approach is more conservative, which may lead to non-significant results.

In this study, the intervention was observed to have the broadest applicability in improving JOA scores, followed by VAS and ODI. This outcome may relate to the different functional aspects reflected by each measure. The JOA score comprehensively reflects overall neurological recovery, including sensory, motor, and bladder functions, and since limaprost acts directly on the nervous system, its efficacy is more consistent across different patient populations. In contrast, VAS is heavily influenced by individual pain perception and inflammatory responses, while ODI is closely associated with postoperative rehabilitation adherence, lifestyle, and muscle function recovery, resulting in greater interpatient variability. It should be noted that some subgroups had relatively small sample sizes, which may lead to unstable regression estimates and wide confidence intervals, thereby increasing the uncertainty of the results. Therefore, the subgroup analyses in this study should be interpreted primarily as exploratory findings and should not be considered confirmatory evidence for clinical conclusions.

This study also has several limitations. First, as a retrospective study, there may be inherent selection bias in the data. Second, the study sample was relatively small, and other potential influencing factors, such as surgeon experience, were not fully accounted for. Third, the follow-up period was limited to 6 months, which precludes assessment of long-term effects (e.g., 1 year or longer) on neural recovery and risk of restenosis. Outcome assessment in this study was not blinded, therefore, there is a potential risk of measurement bias. Finally, pain and functional scores (VAS, ODI, JOA) are subjective measures and may be affected by patients' psychological state and adherence to rehabilitation. Subgroup analyses involve multiple comparisons, which may increase the risk of type I error (false positives); therefore, these results should be interpreted with caution.

## Conclusion

This study found that, compared with UBE surgery alone, limaprost combined with UBE surgery was associated with better postoperative pain outcomes in patients with LSS (VAS as the primary outcome). More favorable outcomes were also observed in secondary outcomes, including functional disability (ODI) and neurological function (JOA). The effect on pain relief was more pronounced at 1 month postoperatively, while the improvement in neurological function was more evident at 3 months postoperatively. However, no time-dependent trend was observed in functional disability improvement. The improvement in imaging-based outcomes was relatively limited. Stratified analysis indicated that the effects of this intervention varied among different patient subgroups, with the broadest applicability observed in the improvement of JOA scores. This suggests that limaprost, as an adjunct to UBE surgery, may provide certain benefits in pain relief and neurological recovery; however, further large-scale prospective studies are still needed to validate these findings.

## Data Availability

The raw data supporting the conclusions of this article will be made available by the authors, without undue reservation.

## Supplementary material

SUPPLEMENTARY FIGURE S1 Love plot of standardized mean differences (SMDs) of covariates before and after propensity score matching (PSM).
