# Supplementary material for: Efficacy of limaprost combined with unilateral biportal endoscopic surgery in the treatment of lumbar spinal stenosis: based on time effects and stratified analysis
Source: Front Surg. 2026 Jun 25;13:1869975. doi: 10.3389/fsurg.2026.1869975 (PMC13346082; doi:10.3389/fsurg.2026.1869975)
Supplement: SUPPLEMENTARY FIGURE S1 — Love plot of standardized mean differences (SMDs) of covariates before and after propensity score matching (PSM). [file Supplementaryfile1.docx]

| **Supplementary Table. 1 Distribution of baseline characteristics between the control and intervention groups after propensity score matching (PSM)** | | | | | | | | | |
| --- | --- | --- | --- | --- | --- | --- | --- | --- | --- |
|  | | **Overall** | | **Control group** | | **Intervention group** | | | **P-value** |
|  | | **n=60** | | **n=30** | | **n=30** | | |  |
| **Age** | | 66 (60-74) | | 67 (60-75) | | 65 (61-74) | | | 0.976 |
| **Gender** | |  | |  | |  | | | 1.000 |
| Male | | 27 (45%) | | 13 (43.3%) | | 14 (46.7%) | | |  |
| Female | | 33 (55%) | | 17 (56.7%) | | 16 (53.3%) | | |  |
| **BMI** | | 24.7 (23.1-26.4) | | 25 (23-26.1) | | 24 (23.2-26.5) | | | 0.971 |
| **Smoking** | |  | |  | |  | | | 0.566 |
| Non-smoker | | 43 (71.7%) | | 20 (66.7%) | | 23 (76.7%) | | |  |
| Occasional smoker | | 12 (20%) | | 8 (26.7%) | | 4 (13.3%) | | |  |
| Regular smoker | | 5 (8.3%) | | 2 (6.7%) | | 3 (10%) | | |  |
| **Drinking** | |  | |  | |  | | | 0.340 |
| Non-drinker | | 42 (70%) | | 20 (66.7%) | | 22 (73.3%) | | |  |
| Occasional drinker | | 14 (23.3%) | | 9 (30%) | | 5 (16.7%) | | |  |
| Regular drinker | | 4 (6.7%) | | 1 (3.3%) | | 3 (10%) | | |  |
| **Retirement** | |  | |  | |  | | | 1.000 |
| Yes | | 50 (83.3%) | | 25 (83.3%) | | 25 (83.3%) | | |  |
| No | | 10 (16.7%) | | 5 (16.7%) | | 5 (16.7%) | | |  |
| **Exercise habit** | |  | |  | |  | | | 0.487 |
| No | | 32 (53.3%) | | 16 (53.3%) | | 16 (53.3%) | | |  |
| Occasional | | 26 (43.3%) | | 12 (40%) | | 14 (46.7%) | | |  |
| Regular | | 2 (3.3%) | | 2 (6.7%) | | 0 (0%) | | |  |
| **Duration of disease (months)** | | 21 (13-28) | | 21 (12-25.7) | | 20 (13-31) | | | 0.878 |
| **Multi-level involvement** | |  | |  | |  | | | 1.000 |
| Yes | | 37 (61.7%) | | 19 (63.3%) | | 18 (60%) | | |  |
| No | | 23 (38.3%) | | 11 (36.7%) | | 12 (40%) | | |  |
| **Affected spinal level** | |  | |  | |  | | |  |
| **L3-L4** | |  | |  | |  | | | 1.000 |
| Yes | | 15 (25%) | | 8 (26.7%) | | 7 (23.3%) | | |  |
| No | | 45 (75%) | | 22 (73.3%) | | 23 (76.7%) | | |  |
| **L4-L5** | |  | |  | |  | | | 1.000 |
| Yes | | 43 (71.7%) | | 22 (73.3%) | | 21 (70%) | | |  |
| No | | 17 (28.3%) | | 8 (26.7%) | | 9 (30%) | | |  |
| **L5-S1** | |  | |  | |  | | | 1.000 |
| Yes | | 8 (13.3%) | | 4 (13.3%) | | 4 (13.3%) | | |  |
| No | | 52 (86.7%) | | 26 (86.7%) | | 26 (86.7%) | | |  |
| **Imaging-based classification** | |  | |  | |  | | | 0.653 |
| Central canal stenosis | | 34 (56.7%) | | 18 (60%) | | 16 (53.3%) | | |  |
| Lateral recess stenosis | | 13 (21.7%) | | 6 (20%) | | 7 (23.3%) | | |  |
| Combined stenosis | | 13 (21.7%) | | 6 (20%) | | 7 (23.3%) | | |  |
| **Supplementary Table. 2 The complete distribution of Modified MacNab criteria and MMT grades in both the control and intervention groups.** | | | | | | | |  |  |
|  |  | | **Control group** | | **Intervention group** | | **P-value** |  |  |
|  |  | | **n=50** | | **n=50** | |  |  |  |
| **T0** | **Manual Muscle Testing (MMT)** | |  | |  | | 0.269 |  |  |
|  | Grade 3 | | 1（2.0%） | | 0（0.0%） | |  |  |  |
|  | Grade 4 | | 47（94.0%） | | 44（88.0%） | |  |  |  |
|  | Grade 5 | | 2 (4%) | | 6 (12%) | |  |  |  |
| **T1** | **Manual Muscle Testing (MMT)** | |  | |  | | 0.004 |  |  |
|  | Grade 4 | | 47（94.0%） | | 35（70.0%） | |  |  |  |
|  | Grade 5 | | 3 (6%) | | 15 (30%) | |  |  |  |
| **T2** | **Manual Muscle Testing (MMT)** | |  | |  | | 0.045 |  |  |
|  | Grade 4 | | 32 (64%) | | 21 (42%) | |  |  |  |
|  | Grade 5 | | 18 (36%) | | 29 (58%) | |  |  |  |
|  | **Modified MacNab Criteria** | |  | |  | | 0.043 |  |  |
|  | Good | | 34 (68%) | | 23 (46%) | |  |  |  |
|  | Excellent | | 16 (32%) | | 27 (54%) | |  |  |  |
| **T3** | **Manual Muscle Testing (MMT)** | |  | |  | | 0.014 |  |  |
|  | Grade 4 | | 26 (52%) | | 13 (26%) | |  |  |  |
|  | Grade 5 | | 24 (48%) | | 37 (74%) | |  |  |  |
|  | **Modified MacNab Criteria** | |  | |  | | 0.007 |  |  |
|  | Good | | 26 (52%) | | 12 (24%) | |  |  |  |
|  | Excellent | | 24 (48%) | | 38 (76%) | |  |  |  |
| **T4** | **Manual Muscle Testing (MMT)** | |  | |  | | 0.002 |  |  |
|  | Grade 4 | | 13 (26%) | | 1 (2%) | |  |  |  |
|  | Grade 5 | | 37 (74%) | | 49 (98%) | |  |  |  |
|  | **Modified MacNab Criteria** | |  | |  | | 0.017 |  |  |
|  | Good | | 17 (34%) | | 6 (12%) | |  |  |  |
|  | Excellent | | 33 (66%) | | 44 (88%) | |  |  |  |

| **Supplementary Table. 3 Operative variables in the control and intervention groups** | | | | |
| --- | --- | --- | --- | --- |
|  | **Overall** | **Control group** | **Intervention group** | **P-value** |
|  | **n=100** | **n=50** | **n=50** |  |
| **Operative time (min)** | 91 (65-141) | 90 (65-138) | 91 (66-141) | 0.741 |
| **Length of hospital stay (days)** | 4 (1-7) | 4 (1-6) | 4 (1-7) | 0.27 |
